# Simulated HRTEM images of nanoparticles to train a neural network to classify nanoparticles for crystallinity

**DOI:** 10.1039/d4na00266k

**Published:** 2024-07-01

**Authors:** Nina Gumbiowski, Juri Barthel, Kateryna Loza, Marc Heggen, Matthias Epple

**Affiliations:** a Inorganic Chemistry, Centre for Nanointegration Duisburg-Essen (CENIDE), University of Duisburg-Essen 45117 Essen Germany matthias.epple@uni-due.de; b Ernst-Ruska Centre for Microscopy and Spectroscopy with Electrons, Forschungszentrum Jülich GmbH 52428 Jülich Germany

## Abstract

Machine learning approaches for image analysis require extensive training datasets for an accurate analysis. This also applies to the automated analysis of electron microscopy data where training data are usually created by manual annotation. Besides nanoparticle shape and size distribution, their internal crystal structure is a major parameter to assess their nature and their physical properties. The automatic classification of ultrasmall gold nanoparticles (1–3 nm) by their crystallinity is possible after training a neural network with simulated HRTEM data. This avoids a human bias and the necessity to manually classify extensive particle sets as training data. The small size of these particles represents a significant challenge with respect to the question of internal crystallinity. The network was able to assign real particles imaged by HRTEM with high accuracy to the classes monocrystalline, polycrystalline, and amorphous after being trained with simulated datasets. The ability to adjust the simulation parameters opens the possibility to extend this procedure to other experimental setups and other types of nanoparticles.

## Abstract 2

Machine learning approaches for image analysis require extensive training datasets for an accurate analysis.

## Graphical abstract

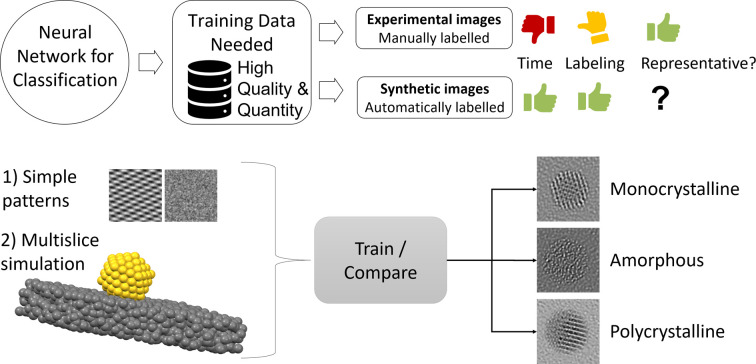

## Introduction

High-resolution transmission electron microscopy (HRTEM) is an important analytical tool in nanoparticle research as size, shape, and atomic structure of nanoparticles are directly reflected in the image contrast. However, analysing such HRTEM images is often a time-consuming and tedious, mostly manual process. A conventional manual annotation can also lead to a considerable degree of human bias in the data processing. Manual analysis is especially limited when large amounts of image data, for instance from *in situ* electron microscopy experiments, are processed. Therefore, automated image analysis, including machine learning (ML), is increasingly used for image analysis tasks in electron microscopy (for comprehensive overviews on machine learning in electron microscopy see ref. 1 and 2). These are typically faster and more objective than manual analyses. For instance, machine learning approaches have been used to perform noise reduction, automated structural analyses of bulk materials in TEM images, as well as the localization of individual atoms and lattice defects.^3–9^ However, large amounts of manually classified training data are necessary to train the neural networks that are used in machine learning.^10–12^

For bulk analyses of HRTEM images of a given sample it is not only of interest to know their size and shape, but also features of their internal structure, *e.g.* to distinguish amorphous, single-crystalline, or polycrystalline configurations. The nanoparticle crystallinity influences their physical properties, *e.g.* their luminescence,^13,14^ their metallic nature,^15,16^ and the stability towards dissolution^17^ which can also effect their biological properties.^18,19^ Notably, a given sample may contain a mixture of nanoparticles with different crystallinity.^20^ In that case, the relative proportions of particles falling into one of these classes are of interest. The principal difference between the three classes of crystallinity is the degree of periodicity of the atomic structure in the particle volume or its projected area, which manifests itself as a corresponding periodicity in the image contrast. The task of classifying samples according to qualitative differences in periodicity in confined areas of an image is a typical task of pattern recognition, which can be performed in real space or in reciprocal space.

Usually, crystallographic analysis is performed by Fourier-transformed HRTEM images and on the electron diffraction patterns on individual particles, for example diffraction using a parallel coherent electron beam.^21,22^ While electron diffraction in cutting-edge microscopes offers the sensitivity to fully characterize a single nanostructure, its success is usually limited to larger features exceeding 3 nm.^23^ With other techniques like X-ray powder diffraction, it is generally difficult to obtain quantitative information on the ratio of amorphous to crystalline particles.^24^ Furthermore, X-ray diffraction averages information over a large number of particles (unlike electron microscopy which probes individual particles), making it blind to variations within smaller clusters or nanoparticles. It also does not give the particle sizes but the averaged size of crystalline domains in a sample. Thus it cannot distinguish between twinned particles and individual particles.^24^

The assessment of crystallinity is particularly challenging when ultrasmall nanoparticles (1–3 nm) are considered.^25^ These are difficult to visualize and conventional electron diffraction is challenging.^26–28^ Furthermore, they are sensitive to internal change (like recrystallization) under the high-dose conditions during electron diffraction.^26,27^ Gold nanoparticles are suitable to address the question of crystallinity because they give a high contrast (unlike the light platinum metals) and because they are not sensitive to oxidation.^26,27^ Thus, gold represents a good role model for ultrasmall nanoparticles and atom-sharp clusters which has been studied to a considerable extent.

We have presented earlier a program based on machine learning to analyse individual nanoparticles for their shape and size from HRTEM images.^29,30^ Here we extend this approach to an automated classification of nanoparticles with respect to their crystallinity. As the generation of manually labelled training data for this task is not only time-consuming but also highly error-prone, different image simulation approaches were tested to establish a feasible training pipeline. This follows earlier approaches to train networks with simulated scanning electron microscopy images for particle size analysis,^7,31^ created by generative adversarial networks (GANs).^32,33^ We present a fully automated classification of nanoparticles by machine learning with respect to their crystallinity, fully based on simulated training data.

## Results and discussion

Even particles with the same crystal structure are usually found in random orientations in a TEM image and therefore can lead to a large number of different patterns in HRTEM images. Thus, a machine learning-based procedure to classify the particles in an HRTEM image-based on their crystallinity is urgently needed. One of the most important factors for a successful machine learning model is the quality and quantity of adequate training data. For a crystallographic classification of nanoparticles, a large amount of accurately labelled HRTEM images is necessary. Manually classified HRTEM images would be ideal, but it is a tedious process to classify thousands of particles. Furthermore, the human bias with respect to the classification of borderline cases immediately affects the quality of the training data.^34^ Therefore, we have investigated approaches applying synthetic HRTEM images to train the neural network. Two different approaches to generate synthetic images were explored, first a simple pattern-based simulation, and second, a more advanced simulation of HRTEM images by the software package “Dr Probe”.^35^ The quality of the classification after training was tested on a subset of the simulated images (test dataset) and also on a manually labelled set of experimental HRTEM images. This ensured that the network was applicable to the experimental images and that the simulated images were an adequate representation of experimental images.

**Fig. 1:**
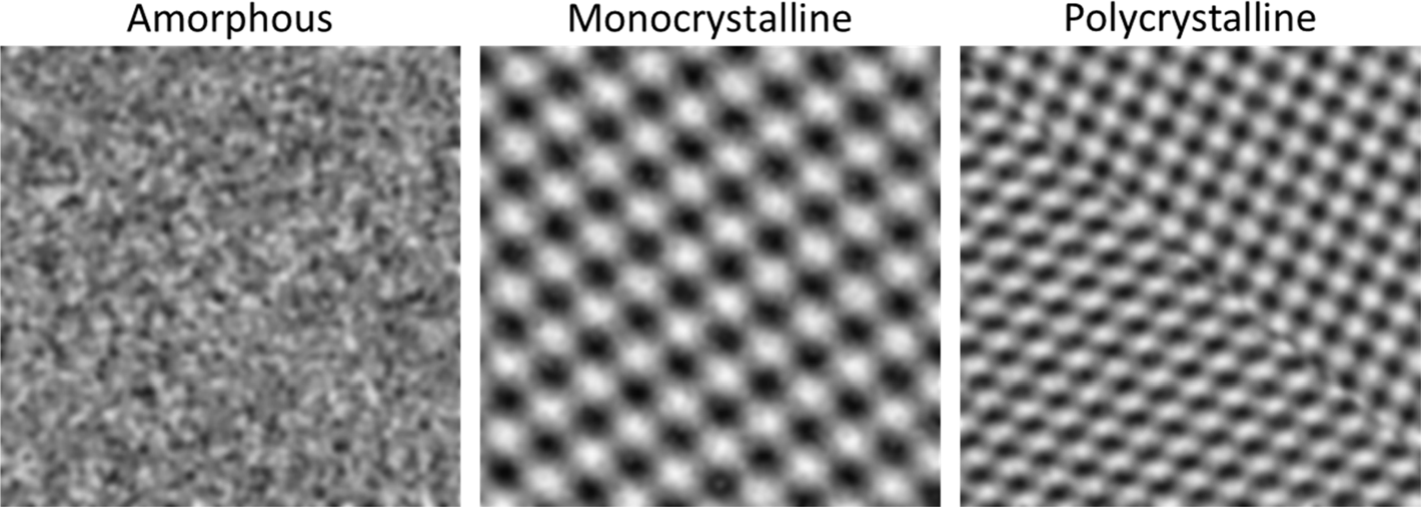
Example images of the simple pattern simulation approach to train a neural network to classify particles into the categories amorphous, monocrystalline, and polycrystalline.

As a first very basic approach, the classification network was trained on simple pattern images as shown in Fig. 1. The training was performed with such patterns without the background signal of the thin amorphous support that is typical for HRTEM images of supported nanoparticles, *i.e.* with the depicted quadratic images.

The training was first performed for two classes (amorphous and crystalline) and then extended to three classes (amorphous, monocrystalline, polycrystalline). The network showed a very good performance on the simulated test dataset which was a subset of 20% of the simulated images that were not used in the training process for the classifications amorphous/crystalline (denoted as “two-class” in the following) and amorphous/monocrystalline/polycrystalline (denoted as “three-class” in the following). However, a test on experimental HRTEM images of ultrasmall gold nanoparticles (1–3 nm) gave disappointing accuracies (Table 1). This indicates that simple patterns are not suitable to train a network to classify experimental HRTEM images.

**Table 1:** Performance evaluation metrics for the network trained on a dataset that consisted of patterned images, tested on the test dataset, and on a dataset of manually labelled experimental HRTEM images of ultrasmall gold nanoparticles

	Class	Simulation test dataset			Experimental HRTEM dataset		
		Accuracy [%]	Precision [%]	Recall [%]	Accuracy [%]	Precision [%]	Recall [%]
Two-class	Amorphous	100	100	100	56.3	28.8	20.7
	Crystalline		100	100		65.0	74.3
Three-class	Amorphous	99.5	100	100	51.74	76.6	59.9
	Mono-crystalline		99.5	99.0		20.6	38.4
	Poly-crystalline		99.0	99.5		47.4	44.9

**Fig. 2:**
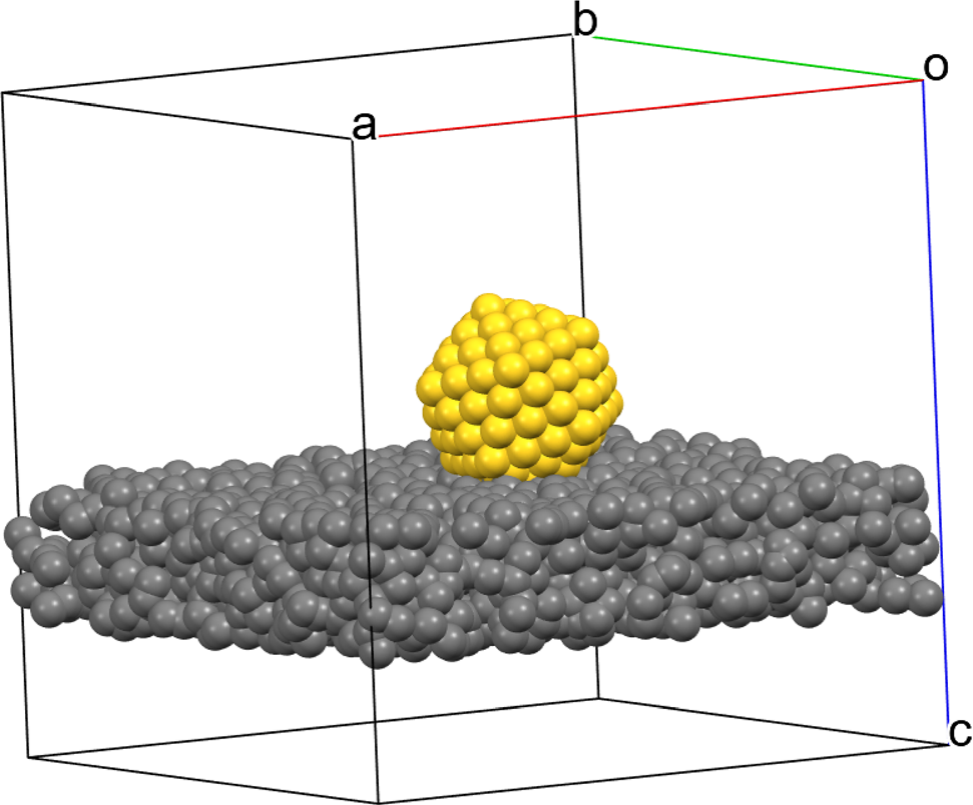
3D model of a gold nanoparticle on a support of amorphous carbon used for the HRTEM image simulation. The edge length of the cubic box is approximately 6 nm. The rendering was performed with the program Mercury.^36^

Simulations of HRTEM images with atomic structure models of gold nanoparticles and thin amorphous support films were performed with the software Dr Probe.^35^ A dataset was created that consisted of simulated images of ultrasmall gold nanoparticles on a support of amorphous carbon as shown by the example in Fig. 2. Gold nanoparticles on a carbon sample holder can be considered as a good model system which is also easily experimentally accessible. Note that we did not consider strict crystallographic structures in this approach, *i.e.* all patterns with a regular pattern indicating a translation symmetry were considered and classified as crystalline.

**Fig. 3:**
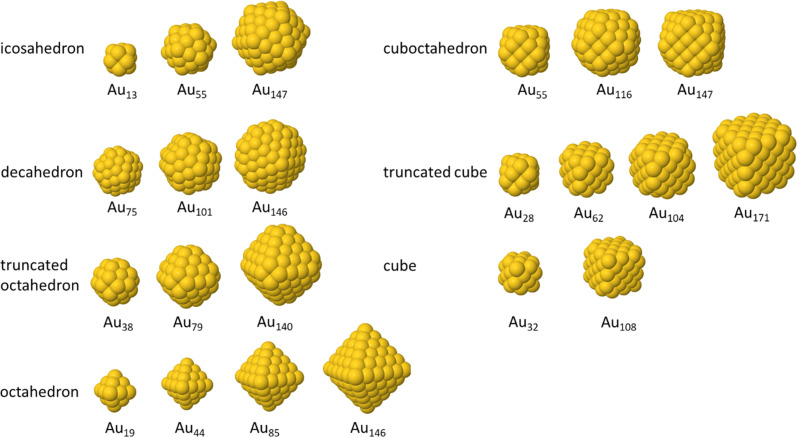
Different types of gold nanoparticles from the ChemTube3D dataset^37^ used for the simulation of HRTEM images.

Different models of gold nanoparticles were used for the simulations, taken from the ChemTube3D database (Fig. 3).^37^ In addition, spherical cut-outs of the gold fcc structure were prepared. Furthermore, amorphous gold nanoparticles were simulated by a custom-made Python script. The presence of amorphous (or disordered) nanoparticles is a peculiarity in the ultrasmall size regime where each particle consists of only a few hundred atoms.^26^

**Fig. 4:**
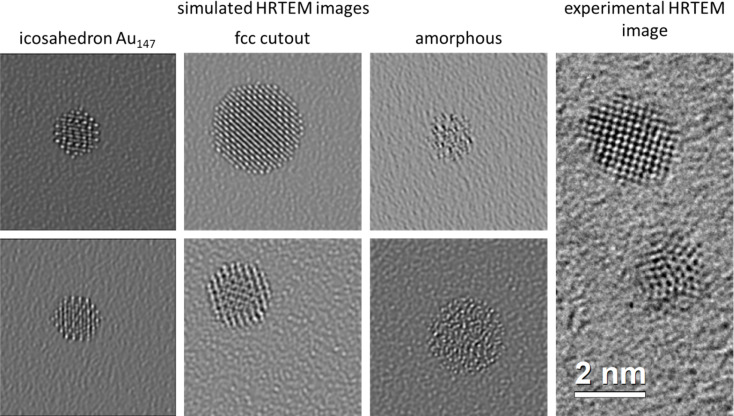
Left: Representative simulated images: two examples of an Au_147_ icosahedron structure from the ChemTube3D database,^37^ two spherical fcc cut-outs, and two examples of generated amorphous particles. Right: A cut-out from an experimental HRTEM image showing two crystalline gold nanoparticles is shown for comparison.

In addition to variations of the structure models, some imaging parameters (including the most volatile optical parameters like defocus and two-fold astigmatism) were varied with each simulation within reasonable ranges. Examples of the simulated HRTEM images are shown in Fig. 4 together with an experimental HRTEM image for comparison. Extensive data augmentation of the primary dataset by rotation, brightness and contrast augmentation, *x*- and *y*-axis rotation, noise addition *etc.* was carried out to increase the number of available training images (see Materials and methods part). Before training on these images, they were processed by the ANTEMA software to separate the particle from the background (cut-out procedure based on machine learning) as described earlier.^30^ An inadvertent inclusion of background into the particle area of interest was therefore avoided. Thus, the training process was kept as similar as possible to the processing of experimental HRTEM images.

The first network trained by a more realistic image simulation by the Dr Probe software was named “SimulationC” and consisted of images based on the ChemTube3D models, spherical fcc cut-outs, and the generated amorphous particles, all on a thin amorphous carbon support. The network was trained to distinguish two classes (amorphous and crystalline) and reached an accuracy of 91.2% on the test dataset and of 75.2% on the dataset of experimental HRTEM images (Table 2). A closer inspection showed that the network was especially error-prone on images with a strong amorphous background signal. The low precision of 60.9% for the class “crystalline” indicates that the network tended to falsely classify crystalline particles as amorphous.

**Table 2:** Performance evaluation metrics for the SimulationC network trained on simulated images of gold nanoparticles on amorphous carbon support, tested on a separate dataset of simulated images and a dataset of manually labelled experimental HRTEM images

Class	Simulation test data			Experimental HRTEM dataset		
	Accuracy [%]	Precision [%]	Recall [%]	Accuracy [%]	Precision [%]	Recall [%]
Amorphous	91.2	85.8	89.6	75.3	84.7	76.7
Crystalline		94.2	92.0		61.1	72.5

**Fig. 5:**
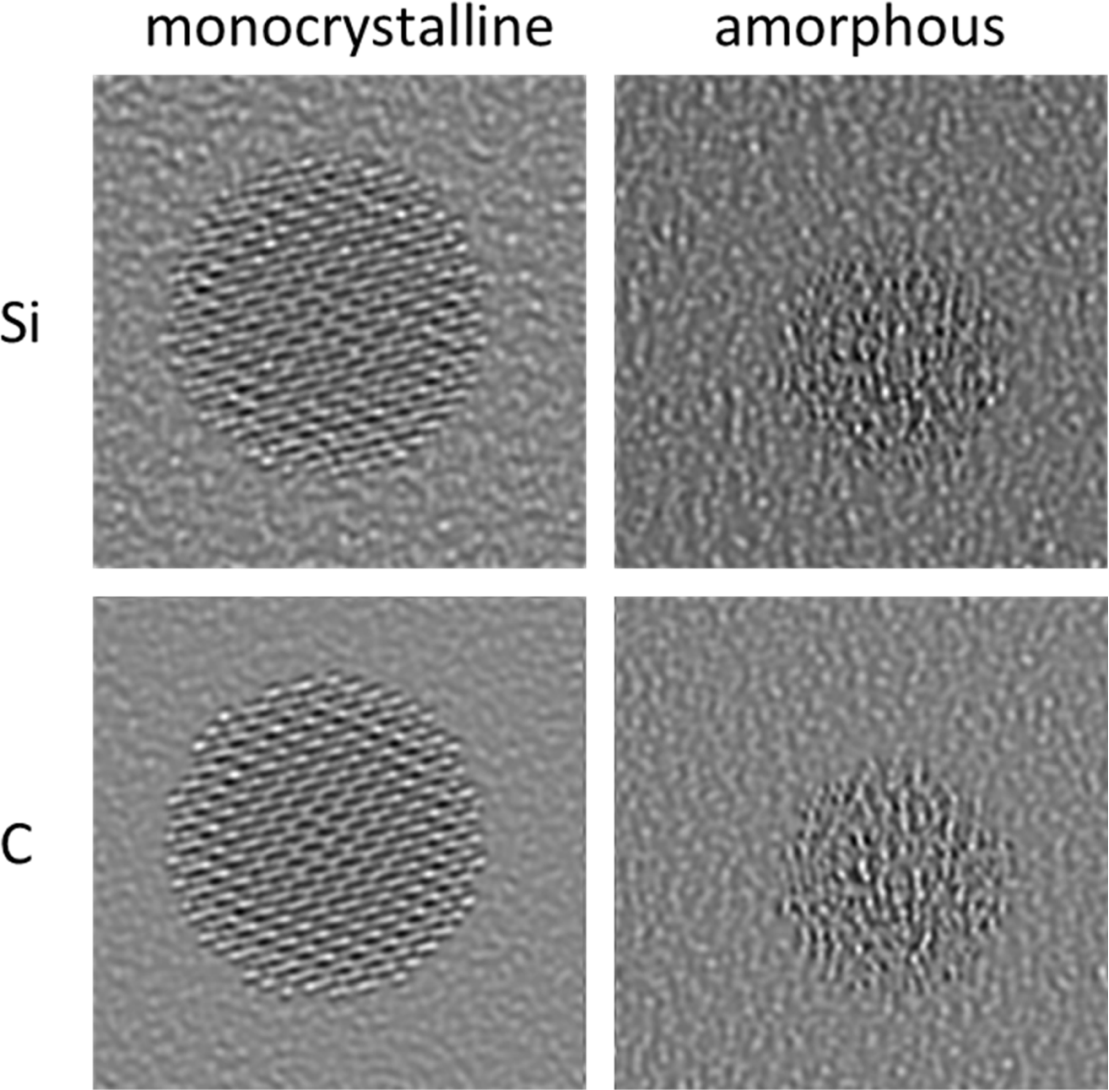
Change of the amorphous support film in the simulation from carbon to silicon. Note the increased contrast of the support film due to the stronger scattering power of silicon compared to carbon.

For this reason, further images were simulated with stronger amorphous background signal. Instead of increasing the thickness of the amorphous carbon film, which would require a serious increase of computation time of the simulation, the background signal was effectively enhanced by preserving the support film thickness, and with this keeping the number of atoms the same but substituting the carbon atoms by silicon atoms. Now the signal of the amorphous background was stronger, reducing the contrast between the background and an amorphous particle (Fig. 5).

The network was trained on an extended dataset that contained the images of nanoparticles from SimulationC and the new nanoparticles on a silicon support. It was denoted as “SimulationC+Si”. For two classes, this network showed a much higher accuracy of 98.7% on the test dataset than the network SimulationC. The accuracy of the network on experimental HRTEM images was also strongly enhanced with 89.3% (Table 3). Obviously, the inclusion of images with stronger amorphous background signals improved the network performance on experimental HRTEM images by generating a more realistic simulation of the level of disturbing background signal.

**Table 3:** Performance evaluation metrics for the SimulationC+Si network trained on of gold nanoparticles on a layer of amorphous carbon and a layer of amorphous silicon support, tested on the simulation test dataset and a dataset of manually labelled experimental HRTEM images

Class	Simulation test dataset			Experimental test dataset		
	Accuracy [%]	Precision [%]	Recall [%]	Accuracy [%]	Precision [%]	Recall [%]
Amorphous	98.7	98.0	99.3	89.3	78.6	93.9
Crystalline		99.4	98.1		96.6	87.1

**Fig. 6:**
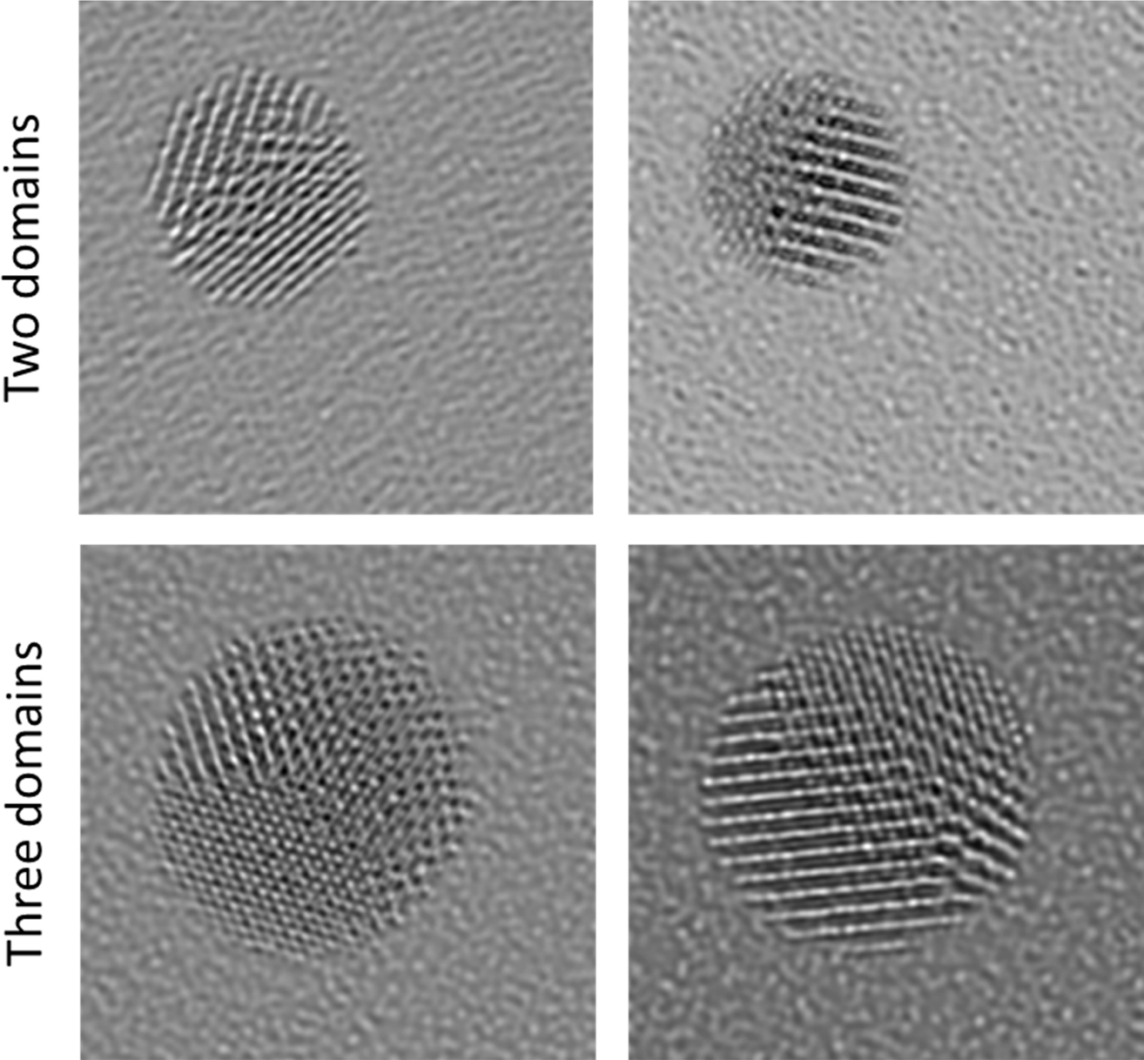
Examples of simulated HRTEM images of polycrystalline particles generated by stitching together differently rotated monocrystalline particles with either two or three different domains. The support in the simulation was silicon in all cases.

**Fig. 7:**
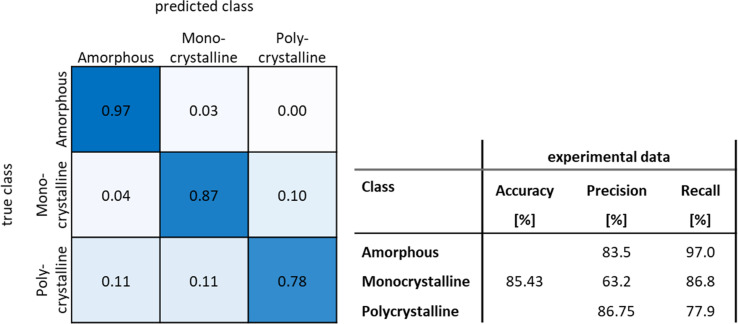
Normalized confusion matrix and performance evaluation metrics for the network “Poly” after omission of all classifications with an accuracy below 80%.

To extend this approach to three classes, simulations of polycrystalline particles were necessary. The polycrystalline particles were simulated on carbon and silicon supports by stitching together either two or three differently rotated monocrystalline fcc cut-outs (Fig. 6). The crystallographic orientation of the domains was not considered. The network trained on this dataset is denoted as “Poly” in the following. This network reached an accuracy of 96.3% on the simulation test dataset for three classes. As might have been expected, errors were mainly made in the distinction between polycrystalline and monocrystalline particles. This was also found with experimental HRTEM test images where the network achieved an accuracy of 78.0%. The main error occurred for polycrystalline particles that were wrongly labelled as monocrystalline, leading to a low precision score of 48.9% for the class monocrystalline (Table 4). After classifications with a low certainty of assignment (<80%) were excluded and categorized as undefined, the accuracy increased to 85.4% and the precision for the class monocrystalline increased to 63.2%. However, this put many particles into the non-assignable category “unknown”. Further errors occurred in the class polycrystalline as shown in the confusion matrix (Fig. 7). The deletion of all classifications with a certainty of assignment below 80% left 19.3% of all particles in the manually labelled dataset categorized as unknown, an acceptable small fraction given that much larger datasets can be evaluated with our automated approach.

**Table 4:** Performance evaluation metrics for the network “Poly”, trained on all data that had been simulated by Dr Probe,^35^*i.e.* from atomic structure models of amorphous, monocrystalline, and polycrystalline gold nanoparticles on amorphous carbon and silicon supports. The performance was tested on the test dataset and a dataset of manually labelled real HRTEM images

Class	Test dataset			Real HRTEM dataset		
	Accuracy [%]	Precision [%]	Recall [%]	Accuracy [%]	Precision [%]	Recall [%]
Amorphous	96.3	99.7	98.2	78.0	81.3	87.9
Monocrystalline		96.4	93.0		48.9	77.7
Polycrystalline		94.2	97.8		92.4	71.8

**Fig. 8:**
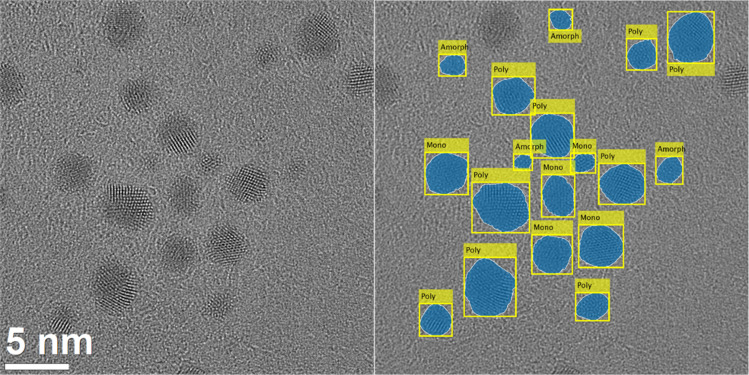
HRTEM image of gold nanoparticles and the combination of the particle detection software ANTEMA^30^ with the particle classification based on crystallinity as implemented here. The particles were classified as either amorphous, monocrystalline, or polycrystalline.

The classification network “Poly” together with the 80% omission rule was then included into the software package ANTEMA^30^ to fully analyse particles in HRTEM images in terms of size, shape and structure. Fig. 8 shows a visualization of the combination of particle detection with ANTEMA and the classification by the network trained with the Poly dataset for an image of gold nanoparticles. The ANTEMA software was able to detect the particles, and the classification algorithm classified the nanoparticles based on their crystallinity. The particles at the border of the image were removed by post-processing in the ANTEMA software to avoid incomplete particles. The analysis by the combined programs took only a few seconds, *i.e.* this approach was much less time intensive than the usual manual analysis. Clearly, the automated analysis gives correct results in most cases. The classification of nanoparticles by size and shape by ANTEMA has been reported earlier.^30^

It should be emphasized that gold nanoparticles represent a particularly good system for this approach because they have a high electron contrast and do not tend to be oxidized.^38^ Therefore, this analysis was possible even for the challenging case of ultrasmall nanoparticles (1–3 nm). This approach will become easier for larger particles (like plasmonic particles), but more difficult for metal particles of lighter elements like silver or the light platinum metals. This is due to the decreasing contrast from these lighter elements that makes the identification of a crystal lattice difficult or even impossible in the ultrasmall particle size range.^26–28^

In principle, it is also possible to analyse crystalline nanoparticles by 2D-Fourier Transformation (2D-FT). This has been demonstrated by Zhu *et al.*^39^ who have applied this method to 7 nm iron oxide nanoparticles. However, the contrast of ultrasmall nanoparticles analysed here is much lower, therefore the analysis will be much more difficult. Furthermore, this is just another method of image analysis, based on training the neural network with 2D-FT images. Therefore, we do not expect a major difference to real-space training as performed here, but this can only be shown in a strict comparison of both methods. It is also an open question how this approach would work on twinned particles that consist of more than one crystalline domain. The current algorithm was designed to cut out individual particles from the image by segmentation. If such a cut-out particle would consist of more than one crystalline domain, Fourier transformation would give erroneous results.

## Conclusions

Ultrasmall nanoparticles with a diameter of 1–3 nm can have an internal crystallinity which is difficult to assess due to their small size, even in high-resolution transmission electron microscopy. Nevertheless, the automated analysis of ultrasmall gold nanoparticles with respect to their crystallinity is possible by application of a suitable machine learning procedure. The necessity for huge training datasets was solved by realistic simulations of HRTEM images of gold nanoparticles on an amorphous support film. Thus, the human bias and the extensive work required by manually classifying thousands of particles can be avoided. The simple approach of pattern-based images was not successful as obviously these patterns are not sufficiently similar to HRTEM images for training. In contrast, HRTEM image simulations can be used to train a neural network for particle classification into the categories amorphous, monocrystalline, and polycrystalline. However, it turned out that small experimental details like the disturbing signal due to the amorphous support film had a strong influence on the quality of the training. This was demonstrated by the significant increase in the level of assignment by changing the support material from carbon to silicon in the simulated images. The simulation is based on specific experimental parameters of the electron microscope used but can in principle be generalized to create any kind of dataset representing a variety of HRTEM imaging conditions. Furthermore, it easily permits to change the chemical nature of the nanoparticle, *e.g.* from gold to other metals or oxides, thus it can be used for different kinds of materials. Of course, the classification of ultrasmall nanoparticles is particularly challenging due to the small number of atoms involved. Consequently, the classification of larger particles should be possible along the same way with even higher accuracy as the periodicity in the HRTEM images is stronger. Further adaptations of the simulation files to produce more realistic images with more noise may further increase the accuracy of a network trained on such simulated data.

In summary, the combination of a particle detection approach with ANTEMA with the particle classification presented here enables an automated large-scale analysis of particle crystallinity from HRTEM images with the possibility of analysing thousands of particles within a few minutes. This strongly speeds up the analysis of samples that would otherwise remain insufficiently characterized and gives a statistically reliable assessment of the properties of a particle population.

## Materials and methods

### Electron microscopy

High-resolution transmission electron microscopy (HRTEM) was performed with an aberration-corrected FEI Titan transmission electron microscope equipped with a Cs-image corrector (CEOS Company), operating at 300 kV.^40^ The nanoparticle dispersion was drop-cast on a copper grid that was coated with an ultrathin amorphous carbon film. Representative TEM images of ultrasmall metallic nanoparticles (1–3 nm) were used for validating the neural networks trained on artificial images.

### Image simulation

Image patterns for the classes amorphous, monocrystalline, and polycrystalline were generated from synthetic patterns with a custom-made MATLAB script.^41^ Images of the class amorphous were generated by placing random black and white dots on a grey background and adding salt and pepper noise with a noise density of 0.5. Images of the class monocrystalline were generated by overlaying two sinus functions with random frequency values in range 0.05 to 0.55 at randomly set angles and adding salt and pepper noise with a noise density of 0.5. Images of the class polycrystalline were generated by stitching together two or three images of the class monocrystalline with the same sinus frequency. All training images were quadratic as shown in Fig. 1.

HRTEM images were simulated with the software Dr Probe, based on a Python interface.^35,42,43^ All generated images depicted gold nanoparticles. The atom packing models were partially acquired from ChemTube3D which are based on calculations by Barnard *et al.*^44,45^ and also generated by dedicated scripts with the tools implemented in the Dr Probe software and the emilys Python package.^37,46^ The data from ChemTube3D provided 16 monocrystalline and 6 twinned models (Fig. 3). Further monocrystalline models were generated by cutting out spheres of random sizes between 1 and 3 nm from the fcc structure of gold (ICSD 52700).^47^ Further polycrystalline particles were generated by cutting two differently rotated monocrystalline spheres of the same size (1 to 3 nm) along the same axes with a random distance from the particle centre between 0 nm and half of the radius of the particle. The first part of the first sphere and the second part of the second sphere were then stitched together to produce a polycrystalline particle. With a 50% chance this procedure was repeated with the resulting polycrystalline particle and another rotated monocrystalline particle of the same size. For this, the polycrystalline particle was randomly rotated before cutting it so that the previous cutting axis and the new cutting axis were not parallel. Amorphous particle models were generated by randomly positioning atoms in a spherical volume and then removing all positions that had a distance to other atom positions below 0.248 nm, following the procedure given by Novaes *et al.*^48^

Each particle was then placed into a cubic box with a side length of about 6 nm with the emilys toolbox.^46^ An amorphous carbon support layer, representing the sample holder, was added below the particle by the same generative approach as used above with the amorphous gold nanoparticles. The filled volume was a cuboid with the length and width of the cubic box and a randomly set thickness between 1 and 3 nm. The minimum distance between the carbon atoms was set to 0.160 nm. The support was generated individually for each simulation, ensuring a variable support structure, a variable support thickness, and a variable background noise in the simulation. To increase the amorphous background signal, images were also generated by replacing the carbon atoms in the support by silicon atoms, leaving all other parameters and atom positions unchanged.

The simulation of particles as depicted in Fig. 2 was performed for an acceleration voltage of 300 kV. The focus spread was randomly set to values between 4.5 and 5.5 nm. The defocus was set to values in the range of −4 to 5 nm. The two-fold astigmatisms in *x*- and *y*-coefficients were independently set to values between −3.0 and 3.0 nm. In total, three different datasets were generated as shown in Table 5.

**Table 5:** Overview of the training datasets generated with image patterns and with Dr Probe after data augmentation, including the number of particles used in each class

Dataset	Description	Amorphous	Mono-crystalline	Poly-crystalline
Pattern	Simple pattern approach with added noise	1000	1000	1000
SimulationC	Simulation with Dr Probe on a carbon support with particle models for crystalline particles created with ChemTube3D and fcc cut-outs	1252	1806	507
SimulationC-Si	SimulationC dataset + simulations with Dr Probe on a silicon support with particle models for crystalline particles created with ChemTube3D and fcc cut-outs	1998	2630	507
Poly	SimulationC-Si dataset + simulations with Dr Probe for polycrystalline (twinned) particles generated from fcc cut-outs on amorphous carbon support as well as on silicon support	1998	2630	3255

### Manually labelled set of HRTEM images

To test the performance of the trained network with real HRTEM data, a set of metal nanoparticles from HRTEM images was manually labelled. These particles were cut out from the images with the ANTEMA software.^30^ This software is based on a neural network trained to distinguish particles from background in HRTEM images by performing semantic segmentation. Thus, individual particles can be detected and isolated from the background. The particles were manually classified as monocrystalline, polycrystalline, or amorphous. Particles that could not be assigned to a class by the examiner with a high certainty were not used for the dataset and excluded from training. The final dataset consisted of 110 monocrystalline particles, 380 polycrystalline particles, and 247 amorphous particles. Chemically, it consisted mainly of gold nanoparticles in a size range of 1 to 10 nm as well as some platinum and silver–platinum nanoparticles for comparison.

### Machine learning procedure

Multiple trainings were performed with different simulated image datasets for training (Table 5) with the goal to assign the particles cut out from real HRTEM images into either two classes (amorphous and crystalline) or three classes (amorphous, monocrystalline and polycrystalline). The datasets generated with the software Dr Probe were pre-processed by treating them with the ANTEMA software to cut out the particle from the image as was done with the real images. This removed the background outside the particle (the support) but not the inherent background noise level caused by the support film below the particle. The patterned datasets were not further pre-processed. All simulated datasets were split into training, validation, and test datasets in a number ratio of 60 : 20 : 20.

Different neural networks that are available in the MathWorks Deep Learning Toolbox were tested for the two-class classification.^41^ The best results were achieved with ResNet-101.^49^ Therefore, this network was used for all further trainings. The weights were initialized with pretrained weights from training with the ImageNet dataset.^50^ As ResNet-101 has an image input size of 224 × 224 pixels, all images were resized to that size. To enhance the training by presenting the network more variable data, extensive data augmentation was applied. The images were augmented by random scaling, rotation, *x*- and *y*-axis reflection, as well as brightness and contrast variation. Furthermore, a random Gaussian filter with a square kernel was applied for image blurring with a maximum Gaussian standard deviation of 2.

The training parameters were optimized by a Bayesian optimization. Training was performed for maximum of 80 epochs. Validation was performed once every epoch to prevent overfitting. If the validation loss did not decrease for more than five validation cycles, the training was terminated. The initial learning rate was set to 0.0085 and decreased every 20 epochs by a drop factor of 0.62.

The computations were performed on a Dell Precision 7920 Tower equipped with an NVIDIA Quadro RTX 5000, 32 GB RAM, and an Intel® Xeon® Gold 6226R processor.

The networks performance was evaluated on the test dataset by the parameters accuracy, precision and recall.^51^ The accuracy is a global metric, defined as the ratio of the correctly classified true positives (TP) and true negatives (TN) to all classified images including the false positives (FP) and false negatives (FN).1
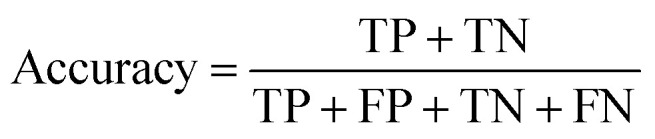


The precision and recall values are class-based metrics. The precision is the ratio of correctly classified images of one class to the full number of images belonging to that class.2
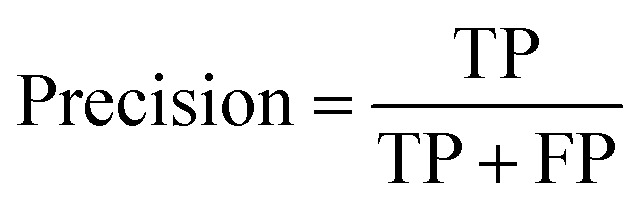


The recall is the ratio of correctly classified images of one class to the full number of images that were classified into this class.3
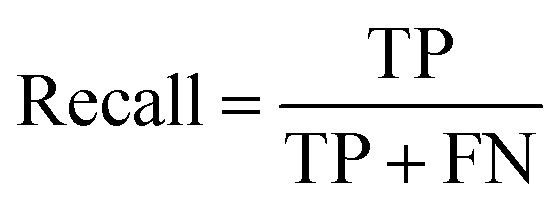


Furthermore, the performance was evaluated on the manually labelled dataset of particles from HRTEM images to test whether the network was applicable to real data.

## Data availability

The code for the described image analysis, denoted with the acronym ANTEMA, including reference images, is available here:

• GitHub at https://github.com/ngumb/ANTEMA.

Further information on the ANTEMA software package has been published here:

• N. Gumbiowski, K. Loza, M. Heggen and M. Epple, *Nanoscale Adv.*, 2023, **5**, 2318–2326.

HRTEM images were simulated with the software Dr Probe, based on a Python interface, as reported here:

• J. Barthel, *Ultramicroscopy*, 2018, **193**, 1–11.

• J. Barthel, Dr Probe command-line tools for HR-(S)TEM image simulation, https://github.com/ju-bar/drprobe_clt, accessed 13.11.2023.

• F. Winkler and E. Julianto, drprobe_interface: Python interface for the Dr Probe command line tools, https://github.com/FWin22/drprobe_interface, accessed 14.11.2023.

The emilys Python package can be found here:

• J. Barthel, emilys: electron microscopy image analysis tools, https://github.com/ju-bar/emilys, accessed 13.11.2023.

## Conflicts of interest

There are no conflicts to declare.

## References

[cit1] Treder K. P., Huang C., Kim J. S., Kirkland A. I. (2022). Microscopy.

[cit2] Botifoll M., Pinto-Huguet I., Arbiol J. (2022). Nanoscale Horiz..

[cit3] Vincent J. L., Manzorro R., Mohan S., Tang B., Sheth D. Y., Simoncelli E. P., Matteson D. S., Fernandez-Granda C., Crozier P. A. (2021). Microsc. Microanal..

[cit4] Zheng H., Lu X., He K. (2022). J. Energy Chem..

[cit5] Sainju R., Chen W. Y., Schaefer S., Yang Q., Ding C., Li M., Zhu Y. (2022). Sci. Rep..

[cit6] Jacobs R. (2022). Comput. Mater. Sci..

[cit7] Ruhle B., Krumrey J. F., Hodoroaba V. D. (2021). Sci. Rep..

[cit8] Groschner C. K., Choi C., Scott M. C. (2021). Microsc. Microanal..

[cit9] Alxneit I. (2018). J. Microsc..

[cit10] Lee B., Yoon S., Lee J. W., Kim Y., Chang J., Yun J., Ro J. C., Lee J. S., Lee J. H. (2020). ACS Nano.

[cit11] Kim H., Han J., Han T. Y. J. (2020). Nanoscale.

[cit12] Ilett M., Wills J., Rees P., Sharma S., Micklethwaite S., Brown A., Brydson R., Hondow N. (2020). J. Microsc..

[cit13] Huang Y. Y., Fuksman L., Zheng J. (2018). Dalton Trans..

[cit14] Sobhanan J., Rival J. V., Anas A., Sidharth Shibu E., Takano Y., Biju V. (2023). Adv. Drug Delivery Rev..

[cit15] Fetzer F., Maier A., Hodas M., Geladari O., Braun K., Meixner A. J., Schreiber F., Schnepf A., Scheele M. (2020). Nat. Commun..

[cit16] Zhou M., Du X., Wang H., Jin R. (2021). ACS Nano.

[cit17] Goudeli E., Pratsinis S. E. (2017). ACS Nano.

[cit18] Skuland T., Lag M., Gutleb A. C., Brinchmann B. C., Serchi T., Ovrevik J., Holme J. A., Refsnes M. (2020). Part. Fibre Toxicol..

[cit19] Gelli R., Ridi F., Baglioni P. (2019). Adv. Colloid Interface Sci..

[cit20] Ruks T., Beuck C., Schaller T., Niemeyer F., Zähres M., Loza K., Heggen M., Hagemann U., Mayer C., Bayer P., Epple M. (2019). Langmuir.

[cit21] Mendoza-Cruz R., Romeu D., Bazan-Diaz L., Samaniego J. E., Santiago U., Ponce A., Jose-Yacaman M. (2017). Cryst. Growth Des..

[cit22] Santiago U., Velazquez-Salazar J. J., Sanchez J. E., Ruiz-Zepeda F., Ortega J. E., Reyes-Gasga J., Bazan-Díaz L., Betancourt I., Rauch E. F., Veron M., Ponce A., Jose-Yacaman M. (2016). Surf. Sci..

[cit23] Bahena D., Bhattarai N., Santiago U., Tlahuice A., Ponce A., Bach S. B. H., Yoon B., Whetten R. L., Landman U., Jose-Yacaman M. (2013). J. Phys. Chem. Lett..

[cit24] Klug H. P. and Alexander L. E., X-Ray Diffraction Procedures for Polycrystalline and Amorphous Materials, Wiley-Interscience, New York, 1974

[cit25] Epple M., Rotello V. M., Dawson K. (2023). Acc. Chem. Res..

[cit26] Wolff N., Loza K., Heggen M., Schaller T., Niemeyer F., Bayer P., Beuck C., Oliveira C. L. P., Prymak O., Weidenthaler C., Epple M. (2023). Inorg. Chem..

[cit27] Wetzel O., Prymak O., Loza K., Gumbiowski N., Heggen M., Bayer P., Beuck C., Weidenthaler C., Epple M. (2022). Inorg. Chem..

[cit28] Wetzel O., Hosseini S., Loza K., Heggen M., Prymak O., Bayer P., Beuck C., Schaller T., Niemeyer F., Weidenthaler C., Epple M. (2021). J. Phys. Chem. B.

[cit29] MacArthur K. E., Polani S., Klingenhof M., Gumbiowski N., Möller T., Paciok P., Kang J., Epple M., Basak S., Eichel R. A., Strasser P., Dunin-Borkowski R. E., Heggen M. (2023). ACS Appl. Energy Mater..

[cit30] Gumbiowski N., Loza K., Heggen M., Epple M. (2023). Nanoscale Adv..

[cit31] Bals J., Epple M. (2023). Advanced Intelligent Systems.

[cit32] Tyagi S., Yadav D. (2022). Arch. Comput. Methods Eng..

[cit33] Goodfellow I., Pouget-Abadie J., Mirza M., Xu B., Warde-Farley D., Ozair S., Courville A., Bengio Y. (2020). Commun. ACM.

[cit34] Bals J., Loza K., Epple P., Kircher T., Epple M. (2022). Materialwiss. Werkstofftech..

[cit35] Barthel J. (2018). Ultramicroscopy.

[cit36] Macrae C. F., Sovago I., Cottrell S. J., Galek P. T. A., McCabe P., Pidcock E., Platings M., Shields G. P., Stevens J. S., Towler M., Wood P. A. (2020). J. Appl. Crystallogr..

[cit37] ChemTube3D , Possible Morphologies of Au Nanoparticles, https://www.chemtube3d.com/aunano_possible-morphologies-of-au-nanoparticles/, accessed 26.10.2023

[cit38] Wagner L. S., Prymak O., Schaller T., Beuck C., Loza K., Niemeyer F., Gumbiowski N., Kostka K., Bayer P., Heggen M., Oliveira C. L. P., Epple M. (2024). J. Phys. Chem. B.

[cit39] Zhu X., Mao Y., Liu J., Chen Y., Chen C., Li Y., Huang X., Gu B. (2023). Nanoscale.

[cit40] Thust A., Barthel J., Tillmann K. (2016). Journal of Large-Scale Research Facilities.

[cit41] MATLAB , The MathWorks Inc., Natick, Massachusetts, 9.11.0.1769968 (R2021b) edn, 2021

[cit42] Barthel J. , Dr Probe Command-Line Tools for HR-(S)TEM Image Simulation, https://github.com/ju-bar/drprobe_clt, accessed 13.11.2023

[cit43] Winkler F. and Julianto E., drprobe_interface: Python Interface for the Dr Probe Command Line Tools, https://github.com/FWin22/drprobe_interface, accessed 14.11.2023

[cit44] Barnard A. S. (2012). Acc. Chem. Res..

[cit45] Barnard A. S., Curtiss L. A. (2006). ChemPhysChem.

[cit46] Barthel J. , emilys: Electron Microscopy Image Analysis Tools, https://github.com/ju-bar/emilys, accessed 13.11.2023

[cit47] Straumanis M. E. (1971). Monatsh. Chem..

[cit48] Novaes F. D., da Silva A. J. R., da Silva E. Z., Fazzio A. (2003). Phys. Rev. Lett..

[cit49] He K. , Zhang X., Ren S. and Sun J., Deep Residual Learning for Image Recognition: 2016 IEEE Conference on Computer Vision and Pattern Recognition (CVPR), 2016, pp. 770–778, 10.1109/CVPR.2016.90

[cit50] Russakovsky O., Deng J., Su H., Krause J., Satheesh S., Ma S., Huang Z., Karpathy A., Khosla A., Bernstein M., Berg A. C., Fei-Fei L. (2015). Int. J. Comput. Vis..

[cit51] Holm E. A., Cohn R., Gao N., Kitahara A. R., Matson T. P., Lei B., Yarasi S. R. (2020). Metall. Mater. Trans. A.

